# Quality of youth friendly sexual and reproductive health Services in West Gojjam Zone, north West Ethiopia: with special reference to the application of the Donabedian model

**DOI:** 10.1186/s12913-020-05113-9

**Published:** 2020-03-24

**Authors:** Alemtsehay Mekonnen Munea, Getu Degu Alene, Gurmesa Tura Debelew

**Affiliations:** 1 School of Public Health, Bahir Dar University, Bahir Dar, Ethiopia; 2 Department of Population and Family Health, Institute of Health, Jimma University, Jimma, Ethiopia

**Keywords:** Quality, Adolescent, Youth, Simulated client

## Abstract

**Background:**

Although there has been momentum in implementing sexual and reproductive health services in Ethiopia, young people remain underserved despite their demonstrated needs. Quality care improves utilization of health service and increases the likelihood of obtaining ongoing care. However, little is known about the quality of youth-friendly sexual and reproductive health service in Ethiopia. Therefore, this study sought to investigate the quality of youth-friendly sexual and reproductive health service in West Gojjam Zone, North West Ethiopia.

**Methods:**

Health facility-based cross-sectional study was conducted in West Gojjam zone in 2018 to assess the quality of the service using the Donabedian model. The assessment was done through the triangulation of multiple methods: simulated client study; structured interviews with service providers; observations; and key informant interview with providers and expertise. Fifty-four visits were made to 18 randomly selected health facilities by three simulated clients trained to present three different scenarios (i.e., adolescent with sexually transmitted infection, pregnancy test request and a lady with dry cough). Data were entered and analyzed using SPSS version 21. Facility visit score of ≥ 75% in all quality component categorized as “good quality” otherwise classified as performing below the standard. Thematic analysis was done to analyze qualitative data.

**Results:**

In this study, none of the health facilities achieved ≥ 75% in the three components of quality measurement. From 18 health facilities, 6(33.3%) provided low quality in all domains. Process component, which measures client-provider interaction and privacy/confidentiality, was the most compromised one. However, a promising result was reported in the input quality that measured the availability of trained providers, drugs, and supplies. The presence of community-based health insurance and age driven comprehensive youth-friendly service delivery approach were identified as challenges to deliver quality services.

**Conclusions:**

The quality of the service ranges from low to medium, with adolescent related elements performing poorly. Minor renovations of health facilities, training on client handling, and contextual modifying the age driven youth-friendly service approach may improve the quality of the services.

## Background

Adolescence, age between 10 and 19 years, is marked as a period of transition and experimentation. This period not only brings changes to their body but also vulnerabilities to the undesirable effect of sexuality, marriage, and childbearing [1]. Globally, pregnancy and childbirth are among the main contributors to diseases and disability among adolescents: early childbearing linked with a higher risk of unsafe abortions, maternal mortality, and morbidity [2, 3]. Teen pregnancy and sexually transmitted infections (STI) can have negative consequences for maternal and child wellbeing, and limit women’s lifelong opportunities, increasing gender and social inequities [2].

Young people in Ethiopia are at risk of a broad range of Sexual and Reproductive Health (SRH) problems such as unwanted pregnancies, unsafe abortion, pregnancy-related complications, and sexually transmitted infections [4–7]. Besides, they have high unmet need for family planning utilization and limited awareness of STI prevention [7, 8].

World Health Organization (WHO) promotes Youth Friendly Reproductive Health Services (YFRHS) to improve the SRH of the young generation. It is highly specialized and a cost-effective program that could contribute to better health among young people through reducing SRH problems such as unwanted pregnancies, new HIV infections, STI and increased overall service utilization [9–12].

YFRHS programs were implemented in Ethiopia since 2006, primarily by NGOs (Pathfinder was pioneer). Currently, it is owned by the government and implemented in an age driven approach in the existing public health facilities [13]. For the effective implementation of the program, standards on YFS, delivery guideline, and minimum service delivery package were developed [14, 15]. By now, the country is implementing the second National Adolescent and Youth Health Strategy (2016–2020). The Strategy goes beyond sexual and reproductive health issue, such as nutrition, mental health, substance use, and injuries. Besides, the government provides comprehensive YFS training to manage all young people in the population (age between10–24) in one room (age driven approach) irrespective of their complaint [16, 17].

This program includes comprehensive SRH services like information and counseling on SRH issues, family planning counseling and methods provision, condom promotion and provision, testing services (pregnancy, HIV), management of sexually transmitted infections, and other medical conditions with appropriate referral linkage [17, 18]. Over 44.7 and 53.5% of health facilities implement YFS program in Ethiopia and Amhara region using an age driven approach, respectively [16, 17]. Additionally, the government is working to scale up YFS in all health centers, hospitals, and university clinics in the same fashion [17, 18].

However, only accessing the services is not enough, quality, as an integral element to optimize the health of young people, should not be neglected. Quality-of-care in adolescent has important implications as a lower quality linked to higher unplanned pregnancy and Sexually Transmitted Infections (STI) rates [19]. Similarly, quality contraception service reduces unintended pregnancies- that would mean averting unplanned births, abortions and maternal deaths [20].

The existing literature focuses merely on assessing factors that affect YFSRH service utilization and quality, focusing on specific dimensions. According to these studies, YFSRH services given at health institutions are less utilized by potential users. Socio-cultural norms, in general, have been identified as factors for poor service utilization [13, 21–26]. So far, little is known about the extent of quality of YFSRH service in Ethiopia. Therefore, the objective of this study was to assesse dimensions of quality of sexual and reproductive health service and generate evidence that would inform health care providers, policymakers, and different organizations to improve youth-friendly services in public health facilities.

## Methods

### Research approach

In this study, the Donabedian model, which is the most common and comprehensive quality evaluation framework- was used. This model defines three distinct aspects of quality: structure (input), process and outcome. Input is defined as the professional and organizational resources associated with provision of health care (e.g. availability of medicines/equipment and trained staff); Process as the things done to and for the patient (eg. client-provider interaction and provider’s skill on procedures) and outcome as the desired result of care provided by the health practitioner. Outcome is the indirect measure of the quality of services and has two divisions. i) technical outcomes, which are the physical and functional aspects of care, such as absence of complications and reduction in disease, disability and death; and ii) interpersonal outcomes, which include attitude and patients’ satisfaction with the care and influence of the care on patient’s quality of life as perceived by the patient [27, 28].

In this study, the structure (input) component was assessed through the availability of supplies/equipments and trained staff related to YFSRH services. The process component mainly focused on client-provider interaction, provider’s skill on physical examination and condom demonstration. And the outcome was focused on interpersonal components such as simulated client’s attitude towards the service and wheather they recommend the service to be used by others.

A simulated client study, where the healthcare provider is not aware that a given client is participating in the research, was used to collect the data. This method helped to decrease the level of bias that may be brought by the presence of an independent observer during the consultation (Hawthorne’s effect). Besides, it removed issues of privacy and confidentiality that may occur with direct observation of the researcher [29–31]. However, difficulties in recruitment and training of simulated clients, and the limitations to the type of information that can be collected by them were reported as significant limitations of simulated clients study [32].

In this study, Midwives were deliberately selected and trained as a simulated client to reduce the limitations raised by other researchers. Health professional simulated clients can reduce defective recall, and captures both the observable and intangible aspects of the caregiving process. They can capture whether the procedures are correctly delivered or not, and can provide the most proximal information on the quality of services than actual clients can do who may be affected by the communication skill of the provider.

### Study design, setting and participants

A cross-sectional mixed method study design was employed to investigat the quality of YFS in West Gojjam Zone, Amhara region, North West Ethiopia. West Gojjam is one of the 15 zones in the Amhara region. It has 13 rural districts and 02 city administrations with 362 and 15 rural and urban *kebeles* (the lowest administrative unit), respectively. From 104 governmental health centers in the Zone, 59 implement YFS program years back [33]. According to the WHO recommendation, 18 facilities were randomly selected and participated in the study [34]. In this study, 18 heads of the health facilities, 18 YFS providers and three simulated clients were involved. Eight key informants comprised of YFS providers, head of the selected health facilities, zonal and regional health bureau officers working in the area were participated in the study. Key informants were selected purposively on the base of their experience on the service, and responsibility on the YFS program.

### Data collection tool

In this study data collection tools were adapt from “Planning, Implementing, and Monitoring for Adolescent and Youth Friendly Reproductive Health Service Standards in Ethiopia”, which is similar to the “World Health Organization Global standards for quality health-care services for adolescents” [19, 35]. The tools were slightly modified inline with the most commonly used indicators for quality aseessment in developing countries (accessibility, staff characteristics and competency, and confidentiality and privacy) [36].

Five data collection tools were used: (1) facility audit checklist: it was used to interview the head of the health center about the availability of essential equipment, drugs, and supplies at the time of the survey, (2) provider’s interview tool: it was used to assess the provider’s overall experience related to the service (eg.; training exposure on case management guidelines and providers’ involvement in providing SRH service at school), (3) Client–provider interaction checklists: it was used to collect information about the service delivery process (eg., the provider’s technical and communication skills, issues of privacy, and confidentiality), (4) client exit interview tool: was used to assess simulated client’s attitude towards the interaction that they made, technical expertise of the providers as well as their recommendation of the service to others, and (5) an observational and record review checklist was used to check whether the health center had YSRH related documents (eg., guidelines), supplies (eg., contraceptives, condoms), overall infrastructure and cleanness of the surroundings. The tools were pre-tested in a non-selected health facility, and minor modification was made to make it appropriate for simulated clients. (See Additional file 1).

### Simulated client preparation

Both male and female graduating class midwifery students’ were recruited from Bahir Dar University, to be trained as simulated clients. They were selected based on their skills in communication and procedures. The training enhanced their ability to collect and document reliable information from their observation and interactions with the providers.

Two gender-specific SRH and one medical complaint scenario were developed. The scenarios focused on Sexually Transmitted Disease (STD), request for pregnancy test and lady with complaint of dry cough. The two SRH scenarios were developed to represent the center of sexual and reproductive health issues that lead the provider to touch all components of the YFSRH service package. In such situations, the health care provider is expected to discuss on adolescents risky sexual behaviors such as number of sexual partner/s and condom use, condom demonstration, other contraceptives, assessment of STI exposure, HIV counseling and testing, or referral if needed [17]. Simulation on cough was deliberately developed to assess the extent of the provider’s actual practice in addressing SRH issue in the case of complaint other than SRH (Table 1).

**Table 1:** Summary of simulated client scenarios

scenarios	Details
Adolescent boy having STI symptoms	- A 19-year-old patient is having urethral discharge, itching, and burning sensation around his genital organ for the last 1 week.
	- He has a history of multiple sexual partners (*the simulated client gave this response when the provider asked him)*
	- He had never used a condom and has no skill to use it *(the simulated client gave this response when the provider asked him, and the simulated client will ask the provider to show condom demonstration)*
A lady who wants to use pregnancy test service A lady with dry cough for 1 week	- A 19-year-old lady who missed her menses for the last 2 months
	- Had nausea and vomiting a week back
	- she has a history of multiple sexual partners (*the simulated client gave this response when the provider asked her)*
	- she does not use any contraceptive methods (*the simulated client gave this response when the provider asked her)*
	- negative test result and she doesn’t want to be pregnant before her marriage
	- An 18-year-old lady came with dry cough for one-week duration
	- She had started a sexual relation (*the simulated client gave this response when the provider asked her)*
	- she does not use any contraceptive methods (*the simulated client gave this response when the provider asked her)*

The national STI management guideline [37] was used to develop the scenarios in consultation with simulated clients. The SCs were trained for 2 days using the YFSRH training manual and simulated client preparation guideline [38]. The training included: condom demonstration, socio-demographic profile of the character, how to present their complaint to the provider and how to complete the tool. They were informed to adhere to their particular scenario and perform it throughout the visited health facilities. Each SC made 18 visits. Besides, they were strictly told to use the local language (Amharic) throughout the interaction made with providers and to be cooperative during physical examinations.

### Data collection procedure

The simulated clients were acting both as a client as well as a data collector. Both client-provider interaction and client exit interview checklists were completed by them. Data were collected after getting approval from the heads and consent from the providers. During the approval process, providers were informed about simulated client study and a health facility visit in any of the days (without telling the exact date). The actual data were collected after a month of the consent. Attempts were made by the simulated clients to avoid any transfer of bias. Their wording and dressing style were similar to the surrounding dwellers. Immediately after they leave the visited health facility compound, the simulated clients completed the checklists. Interviews with heads and providers, observation of the surrounding and document review, and key informant interview data were collected by the authors at the date of the consent.

### Measurement and operational definitions

A total of 103 items which had a ‘yes/no’ response was used to assess the quality of AYFS. Input (eg., Are health workers providing services to adolescents trained on YFS?), process (eg., Did the service provider introduce himself/herself to the client?) and outcome (eg., Will you recommend the health facility to be used by others?) quality were measured using 47, 41, and 15 items respectively. The internal consistency (Cronbach’s alpha) of the items were 0.75, 0.86 and 0.82 for input, process, and outcome, respectively. The UNFPA approach, which is commonly used in developing country, was used as a benchmark to categorize health facility for its quality [39, 40]. Facilities leveled as “good” quality in input, process, and output if it scored at least > 75% of the items intended to measure the respective quality components. “Medium” if scored 50–74% of the items, and low quality was < 50% for each quality components. Facility visit score of *≥*75% in all quality component categorized as “good quality,” otherwise classified as performing below the standard.

### Data management and analysis

The data were entered and analyzed using SPSS version 21. The items disaggregated in its respective quality components by using “*TOOLs (Planning, Implementing, and Monitoring) for Adolescent and Youth Friendly Reproductive Health (AYFRH) Service Standards in ETHIOPIA”* [35]. The unit of analysis was health facilities. The response of each item was coded 0 for “No,” and 1 for “Yes” and equal weight was given for each response. In items which required being assessed using both observation and interview method, the observed responses were considered. The score for each item was calculated based on the number of responses given from the simulated clients. When two of the simulated client answered “yes” for a given item, then that item was scored as ‘one’ otherwise ‘zero.’ Descriptive and thematic analyses were done for quantitative and qualitative data, respectively.

### Ethical consideration

Ethical clearance was obtained from the Institutional Review Board (IRB) of Bahir Dar University, college of medicine, and health sciences. Participants were informed about their right to refuse or discontinue participating in the study. All data were given an identification number and were anonymously processed. Regarding the use of the simulated client method, permission from the head of the health facility, and consent from the health care provider were taken without informing the exact date of health facility visits. Informed consent was taken from simulated clients to participate in the study.

## Results

### Background characteristics of health facilities

In 18 health facilities, a total of 36 health care providers and three simulated clients (made 54 visits) and eight key informants participated in the study. Nearly 2/3 of health facilities serve for more than 25,000 populations, and almost all of the facilities started to provide YFSRH service for about a year a go. The majority had only one trained provider on YFSRH services (Table 2).

**Table 2:** Frequency distribution of health facilities by its background characteristics in West Gojjam Zone, Ethiopia, May 2018

Background characteristics	Frequency	Percent
Catchment populations		
< 25,000	5	27.8
*≥*25,000	13	72.2
Number of health care providers		
< 10	3	16.7
*≥* 10	15	83.3
Duration of YFS		
1–2 year	17	94.4
> 2 year	1	5.6
Number of YFSRHS trained person		
0	4	22.2
1	11	61.1
≥ 2	3	16.7
Availability of separate room for YFS		
Yes	10	55.6
No	8	44.4

### Quality status of health facilities

Taking the overall quality of services into account, none of the health facilities were providing good quality of services (*≥*75%). Twelve (66.7%) and 6(33.3%) health facilities were providing medium and low quality of services, respectively. Taking the three quality indicators separately, 11(61.1%) health facilities and only one facility had good quality of input and outcome performances, respectively. None of the health facilities had good quality of process performance. Fourteen (77.8%) health facilities had low quality of process performance (Table 3).

**Table 3:** Frequency and percentage distribution of health facilities according to the Quality components in West Gojjam Zone, North West Ethiopia, May 2018

Quality components	Good (*≥* 75%)	Medium (50–74%)	Low (< 50%)
Input	11 (61.1%)	7 (38.9%)	0 (0%)
Process	0 (0.0%)	4 (22.2%)	14 (77.8%)
Outcome	1 (5.6%)	7 (38.9%)	10 (55.6%)
Overall quality^a^	0 (0%)	12 (66.7%)	6 (33.3%)

^a^ Overall quality was calculated based on the status of the 3 components of quality of care

The distribution of items responded positively were varied among quality components. The greatest failure was observed in process measuring items followed by the outcome. On the other hand, input measuring items were found better in all health facilityes. From 47 items included in this domain 18(38.3%) were scored by all health facilities. However, nearly a quarter of process measuring items was not fulfilled by many health facilities (Table 4).

**Table 4:** Frequency distribution of the number of items scored by the health facilities, West Gojjam, North West Ethiopia, May 2018

***Quality components (total items)***	Items scored by all health facilities	Items scored by *≥* 75% of the facility	Items not scored by any of the facilities
Input (47 items)	18 (38.3%)	33 (70.2%)	1 (2.1%)
Process (41 items)	4 (9.8%)	11 (26.8%)	9 (22%)
Outcome (15 items)	1 (6.7%)	1 (6.7%)	3 (20%)
Overall (103 items)	23 (22.2%)	45 (43.7%)	13 (12.6)

#### Quality items not fulfilled by any health facilities

None of the health facilities had a signpost containing information on working days and hours for the provision of SRH services. Also, no health facility provided information on the available SRH services to clients. Besides, providers didn’t take time to listen and do the necessary examination and deliver the services (Table 5).

**Table 5:** List of quality items not fulfilled by any of the health facilities, West Gojjam, North West Ethiopia, May 2018

Quality components	Items not achieved	Quality assessment items
Input (47 items)	1 (2.1%)	Presence of signpost containing information on working days and hours for the provision of SRH services
Process (41 items)	9 (22%)	Did the service provider used tools for planning, implementation, and monitoring of YFS
		Did the service provider participated in delivering information on adolescent SRH rights and needs in Youth centers
		Did the service provider participated in delivering information on adolescent SRH rights and needs in Adolescent and or youth meetings
		Did the service provider receive regular guidance on psychological, physical assessment and individualized care on adolescent & youth health services?
		Did the service provider use two-way referral forms when referring to adolescents & youth to other services?
		Did the service provider introduce himself/herself first to the adolescent?
		Did the service provider assure the client that no information will disclose to anyone (parents/other) without their permission?
		Did the service provider ask the adolescent questions about home and relationships with adults?
		Did the service provider use audio-visual material to explain anatomy, disease, or others, as relevant to the topic of the consultation?
Outcome (15 items)	3 (20%)	Did the client receive information on the available SRH services in this health facility?
		Did the client believe the information that he/she provided kept in secret (confidential)?
		Did the provider take time to listen, do the necessary examination, and deliver the services?
Total (103 items)	13 (12.6%)	

### Selected process and outcome measuring items related to communication, privacy, and confidentiality

None of the providers introduced themselves to simulated clients to build a good rapport. Only in 7(38.9%) health facilities, providers asked about their psychosocial history (schooling and substance use), and in 7(33.9%) health facilities, providers listened to simulated clients with attention. Physical examination of simulated client was done in 11(66.1%) health facilities; only in 6(54.5%) health facilities, clients were asked permission before physical examination by the provider. Clients who discussed how to prevent diseases, and what to do to stay healthy were only in 10(55.6%) health facilities. However, in all health facilities, simulated clients were asked about their sexual relations (Table 6).

**Table 6:** Frequency distributionof health facilities performing selected process measuring items, West Gojjam Zone, May 2018

	Health Facilities	
	Number	%
**Selected items related to communication**		
Did the service provider introduce himself/herself first to the adolescent?	0	0
Did the service provider use audio-visual material to explain anatomy, disease, or others, as relevant to the topic of the consultation?	0	0
Did the service provider check the adolescence client’s understanding of the information provided by asking probing questions?	1	5.6
Did the service provider listen with attention to what the client had to say?	6	33.3
Asked the adolescent questions about school, smoking, alcohol, or other substances?	7	38,9
Do physical examination (specific to the complaint)?	11	61.1
Did the service provider explain the results of the physical examination of the client?	8	44.4
Provide accurate and precise information on the management/treatment options?	4	22.2
Ask the adolescent client’s permission before performing the examination/procedure?	6	33.3
Inform the adolescence client about the services available for him/her?	7	38.9
Talk about how to prevent diseases, and what to do to stay healthy?	10	55.6
Provide accurate and precise information on the medical condition?	4	22.2
Asked the adolescent questions about sexual relationships?	18	100
**Selected items related to privacy and confidentiality**		
Did the provider mad an attempt to ensure both auditory and visual privacy (closed door)?	5	27.8
Did anyone present at all the time in the room during your consultation?	3	16.7
Did the service provider assure the client that no information will disclose to anyone?	0	0
Did anyone else enter the room during the consultation (multiple interruptions)?	4	22.2

Regarding privacy, none of the providers assured clients about confidentiality issues. Lack of confidentiality was demonstrated in more than half of the visited health facilities. Inconvenient physical setup (the majority were adjacent to outpatient department), presence of multiple interruptions and leaving doors open during consultation, were the most critical reasons for lack of confidenciality identified by simulated clients (Table 7).

**Table 7:** Frequency distribution of health facilities performing selected output measuring items, West Gojjam Zone, May 2018

Selected interpersonal outcome measuring items related to communication, privacy, and confidentiality	Health facilities	
	Number	%
Do you believe the information you provided kept in secret (confidential)?	0	0
Did the provider take time to listen, do the necessary examination, and deliver the services?	0	0
Did you find the health facility comfortable (location of the room)?	5	27.7
Do you believe that others could not see your consultation with the care provider?	5	27.7
Was she/he critical of any of your words or actions?	6	33.3
Did the health-care provider listen to what you said with interest?	8	44.4
Did the health-care provider treat you in a supportive and considerate manner?	8	44.4
Do you believe that others could not hear your discussion with the health-care provider?	8	44.4
Did you get medicines and supplies for you at this facility?	18	100
Will you recommend the health facility for others?	3	16.7

Health facilities were also categorized in terms of the proportion of quality achievement. The result indicated that only one health facility (5.6%) had good quality in two of the three quality components (achieved 2/3). Whereas, 7(38.9%) health facilities didn’t have good quality in any of the three quality components (achieved 0/3); and the majority, 10 out of 18, health facilities met one out of the three quality domain.

### Status of health facilities by the key quality issues recommended by the ministry of health of Ethiopia

**Fig. 1:**
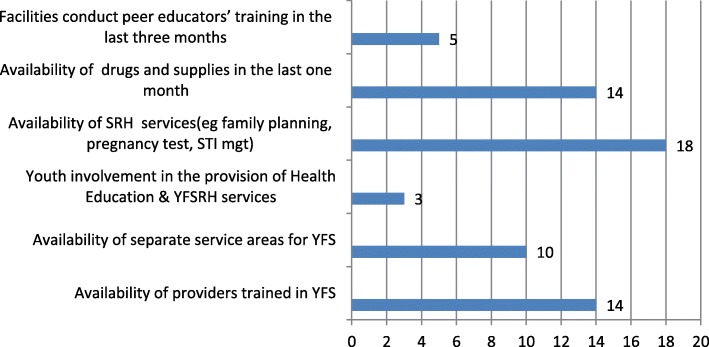
Frequency distribution of health facilities by the key quality issues recommended by the Ministry of Health of Ethiopia, West Gojjam Zone, North West Ethiopia, May 2018

All SRH services (such as family planning, pregnancy test, abortion service, and HIV counseling and testing) were available in all health facilities in the last 1 month preceding the survey. Fourteen health facilities (77.8%) had trained providers. Only 3(16.7%) health facilities involved youth in the provision of YFRH services (information) in the school/community (Fig. 1).

### Qualitative findings

The qualitative data were collected to explor the gap that may not have been addressed by the quantitative data. The in-depth interview mainly focused on providers experience in providing sexual and reproductive health services (eg., the extent of discussing SRH issue), challenges that they faced, and suggestions that may improve the quality of YFS. A total of eight informants (three officers and five YFS providers) participated in the interview. Informants’ age ranged from 23 to 42 years with mean age 28.6 ± 5.9 SD and the majority had more than five years work experiences. More than half of the informants were degree holder and took YFS training.

High client fllow was experienced in all YFS providers, and was a reason to close the service delivery point in a health center. Community-based health insurance and age driven YFS service were the primary reasons explained by participants. The officers also supported the providers complaint. They acknowledge that, health service utilization is increasing following the implementation of community-based health insurance. Hence the client flow is obviously increasing and becoming a challenge for providers.

Providers asked about the extent of discussing SRH issue with the clients/patients while they are working in the room. All of the providers clearly explained that the services are delivered based on the chief complaint that the patients had. SRH service providers gave little information to clients/patients who seek services for the benefit of time.

A 24 year health care provider said that “ … *YFS room closed for a few weeks because of the challenge that I faced. I examined more than 60 clients/* patients *per day. It is because of the system that we implement. Within this context providing information or services other than their complaint is impossible at all … ”*.

An informant also supported the above idea and said, “ *… getting more than four clients to visit the YFS room who came from a single household is the usual experience that we face … .. let alone to discuss SRH issues with clients; we did not get enough time to serve them based on their chief complaint. We usually spend extra time in the room to finish the cards. Generally working in this room is tedious and boring*”.

Staff turnover, absence of a dedicated room for YFS, and client/patient load was identified as a challenge by almost all participants. Increasing the number of trained providers, frequent supervision, and constructing extra rooms was recommended by the participants to improve the quality of YFS. Moreover, charge-free service and using extra service hours including weekends was suggested by the officers.

## Discussion

The second National Adolescent and Youth Health Strategy (2016–2020) of Ethiopia is believed to transform the health of the young generation through quality YSRH services [41]. This study assessed the quality of YSRH services provided in health facilities in West Gojjam Zoe, North West Ethiopia. Our findings indicated that the quality of AYFS ranged from low to medium, with key elements performed poorly. Also, significant differences were observed between quality components. Health facilities showed good quality of input component score. However, issues specific to adolescent services were not in line with the standards. For example, none of the health facilities display YFS working hours, no one uses audio-visual material specific to SRH, and only a few of the facilities conduct peer educators’ trainings. Furthermore, about nearly a fourth of the facilities are providing YFS without having a trained provider.

The highest score in the input component in this study may not be associated with the implementation of YFS program; instead it reflects the general requirement to functions as a health care facility as a whole. Furthermore, this is an essential component for maternal health care services -which is the top priority of the country [42]. Related to issues specific to adolescent services, this finding implies that provision of quality of YFSRH is still a significant problem, and the health system has been inefficient in fully adhering to YFS standards. Providing the service within this context can compromise the quality of the service, consequently, affect the health of adolescent.

Studies indicated that socio-cultural context surrounding adolescent sexuality, norms and taboos (leading them to fear and feel shame) were the most discouraging to access SRH services and also has an effect on the quality of services [23]. Adolescents would feel more comfortable if they are attended to in a separate room. Similarly, a study conducted in Mixico showed that a physical space allowing privacy during counseling is an essential element of quality sexual and reproductive health care for adolescents [43]. However, only 10(56.8%) health facilities in this study had a dedicated separate room for youths. As a result, lack of privacy was demonstrated in more than half of the visited health facilities. Specifically, the finding of this study indicated that inconvenient physical setup (the majority were adjacent to other service outpatient department), presence of multiple interruptions and leaving doors open during consultation, were the most critical reasons for pooy privacy. Moreover, in facilities with more than one YFS trained providers, two clients were served at a time in the same room.

The above finding inplies that if services do not provided privacy, clients may not discuss sensitive issues or may contribute to the patients’ ability to fail to disclose concerns on SRH [44]. Furthermore, lack of privacy may affect user satisfaction and their recommendation of the service to others, and even they may not return back again to the service [21, 23].

In this study, though more than ¾ of health facilities have trained providers, most of them were relied on “business as usual” in performing their activities. Clients were treated only based on the complaint that they had. Providers were not satisfied in working in YFS room. They explained that the comprehensive service delivery approach and the existence of community-based health insurance increased their workload and made their work tedious.

This context may be one of the reasons that providers did not take time to listen, to do the necessary examination, and deliver the services as to the standard. For example, none of the simulated clients received information on the ranges of SRH services available in the facilities. Only in 10(55.6%) health facilities, providers discussed about how to prevent diseases and what to do to stay healthy. Even when adolescents’ seek health services for their SRH illnesses, opportunities were lost to engage them in related services like HIV testing. This represents critically missed opportunities to diagnose youth who may live with HIV and reduce onward transmission and it may affect the plan to reaching the 90–90–90 targets set by Joint United Nations Program on HIV/AIDS [45]. This should have critical implications not only for the individual but also for public health and prevention of the diseases in general. Low rates of HIV diagnosis and treatment initiation among young may continue to be a significant challenge to the epidemic control.

Besides, when sessions with health care providers have rushed, the providers may not spend adequate time in asking fundamental questions related to sexual history. Rushed sessions may have an impact on the client’s needs, provide enough information, or adequately answer their questions or concerns [46]. In this case the client may experience the service as poor; and consequently influence client satisfaction negatively. It may further lead to poor service utilization and the SRH problem may continue as a vicious cycle.

The existence of community health insurance appears to be an excellent approach to increase the health-seeking behavior of young people. It creates an environment to contact with many clients on a daily base, and it is an excellent option to capture and equipp adolescent to the necessary information/services on SRH. However, providers did not view this initiative as a good opportunity, rather a cause for a challenge to provide quality service as it makes them feel overburdened. The providers complained that households enrolled in community health insurance are treated at minimal costs, hence hosehold members accompanying an individual with sickness wanted to consult providers without any apparent illness. Such practices increased the work load and depleted the existing meager reasources in the facilities.

Almost in all health facilities, the simulated clients were treated only based on the complaint that they presented. Treating based on clients complaint only may imply that the current service delivery approach (age driven comprehensive service) of YFS may not be promising in addressing the diverse needs of clients. Besides, clients/patients who come from a single household should also be viewed as an opportunity to provide group counseling/education on SRH that may accelerate to break the SRH communication barriers within a family. This may consequently increase YFS service utilization and reduce SRH problems anong adolescents.

### Applicability of Donabedian model

In this model, it was postulated that, there are relationships between input, process, and outcome constructs based on the idea that good structure should promote good process and the good process should, in turn promote good outcome [47]. This can be used to draw inferences about the quality of health care [48]. However, significant discrepancies were identified between input and process component in this study. The findings imply that only good input might not be a guaranty to ensure a good process and bring good outcome. There may be other factors contributing to poor process and outcomes that cannot be addressed by using Donabedian’s model. For example, communication, coordination, accountability, and morale within the organization may be a factor-which was not part of this model. Therefore, the authors would like to recommend future researchers to consider organization-related factors in health care quality assessment.

### Limitations of the study

In this study, one of the limitations that we faced was assessment tool related. Some items used as quality indicator had an ambiguous meaning. For example, ‘signpost present/absent?’ ‘lists of SRH services present/absent?’ may incur the issue of privacy. Hence, some facilities removed or modified the name of the room intentionally to insure privacy issue. The other limitation was on observable vs interview method of data collection items: for example, “ask and observe whether the provider follows the delivery guideline.” In such items, the data collector may record as ‘no’ if he/she is not observing the provider while using the guideline. In reality, the provider may not use the guideline in each instant if he/she understood what is found in it. Such ambigious items may underestimate the proportion of health facilities that provide quality YFSRH services.

Additionally, the measurement of client satisfaction and influence on patient’s quality of life as perceived by actual client/patient were not investigated in this study. Using simulated clients may not give a full picture of the outcome indicators. Therefore, the authors would like to recommend future researchers to use both real as well as simulated clients for better exploration of the of health service quality.

Finally, in this study the name of the participating health facilities and staffs were strictly anonymized to avoid any repercussions on them. However, by anonymizing the health facility we may have overlookd some critical facility-specific findings which may help to future improvement.

## Conclusion

This study indicated that inputs for SRH services such as essential drugs, equipment, and providers are available in health facilities. However, the quality of YFSRHS in the health facilities were minimal. The performance indicators ranged from low to medium, with adolescent related elements performing poorly. The comprehensive approach of YFS –all youth in one room- combined with the existence of community-based health insurance brings the health care provider in contact with many potential youth clients daily. This working context resulted in increased work load at the YFS room thereby making the provider bored and didn’t give attention to SRH issues. The findings suggest that the current approach of YFS is not promising in addressing the diverse and evolving needs of clients and failed to ensure the prioritization of SRH services at health facilities. Vividly, the results indicate that the health system is inefficient in providing the service as to YFS standards.

### Recommendations

The quality of YFS in this study was minimal and mainly can be attributed to process components of YFS provision. Thus, the regional health bureau should consider to to provide in-service capacity building training specifically on client handling and communication skills.

The presence of community health insurance increased the clients/patients flow to the health facilities resulting in many users from a single household. This presents a “never-before” opportunity to address youth SRH. Therefore the Ministry of Health of Ethiopia needs to modify the service delivery approach to address this missed opportunity. This may be achieved through minor renovations of the health facilities and service delivry process within the existing system (e.g. assign two health care providers-one for the complaint and the other for SRH- at the same time using single partitioned room). There is also a possibility of providing group SRH education for those families who came from single households (an innovative one). This approach may encourage discussion on SRH within a family and stimulate community engagement on the issue.

## Supplementary information

**Additional file 1.** Questions for facility review.

## Data Availability

The datasets analyzed during the current study are available from the corresponding author on reasonable request.

## Abbreviations

YFSRH: Youth friendly sexual and reproductive health
YFS: Youth friendly services
SCs: Simulated clients
SRH: Sexual and reproductive health
STIs: Sexually transmitted infections
WHO: World Health Organization
