# Supplementary material for: Quality of youth friendly sexual and reproductive health Services in West Gojjam Zone, north West Ethiopia: with special reference to the application of the Donabedian model
Source: BMC Health Serv Res. 2020 Mar 24;20:245. doi: 10.1186/s12913-020-05113-9 (PMC7092526; doi:10.1186/s12913-020-05113-9)
Supplement: Supplementary file 1 — Additional file 1. Questions for facility review. [file 12913_2020_5113_MOESM1_ESM.docx]

### Additional file 1.

### Questions for facility review

Name of Health facility:__________________ Name of woreda____________________ duration of YFS ----------- catchment population----------youth population------------ number of health care providers ---------------------

**1. QUESTIONNAIRE FOR THE HEAD OF THE HEALTH FACILITY**

| Sn | Question | | | | | | | | | | | | | | | | | | | | | | Response | |
| --- | --- | --- | --- | --- | --- | --- | --- | --- | --- | --- | --- | --- | --- | --- | --- | --- | --- | --- | --- | --- | --- | --- | --- | --- |
| 1 | Does the health facility have the documents, if yes show documents | | | | | | | | | | | | | | | | | | | | | | yes | no |
|  | 1.Standards on Youth Friendly Reproductive Health Services | | | | | | | | | | | | | | | | | | | | | | yes | no |
|  | 2.Tools for planning implementation and monitoring of Standards | | | | | | | | | | | | | | | | | | | | | | yes | no |
| 2 | Are health workers providing services to adolescents trained on YFS? | | | | | | | | | | | | | | | | | | | | | | yes | no |
| 3 | Please tell me if information has been delivered on ASR health needs and available services in the following places during the last one month | | | | | | | | | | | | | | | | | | | | | | yes | no |
|  | 1. Schools | | yes | | | | | | no | | | | | | 3 | | | | | | Community meetings Kebele, Idir meetings | | yes | no |
|  | 2. Youth centers | | yes | | | | | | no | | | | | | 4 | | | | | | Adolescent and/or youth meetings | | yes | no |
| 4 | Please tell me if information has been delivered on ASR health rights in the following places during the last one month | | | | | | | | | | | | | | | | | | | | | | yes | no |
|  | 1. Schools | | | yes | | | | | | no | | | | | 3 | | | Community meetings (e.g. Kebele, Idir meetings) | | | | | yes | no |
|  | 2. Youth centers | | | yes | | | | | | no | | | | | 4 | | | Adolescent and/or youth meetings | | | | | yes | no |
| 5 | Does the health facility have a sign post? containing information on: | | | | | | | | | | | | | | | | | | | | | | yes | no |
|  | 1. the types of adolescent and youth reproductive health services provided? | | | | | | | | | | | | | | | | | | | | | | yes | no |
|  | 2. working days and hours for the provision of AYFRH services ? | | | | | | | | | | | | | | | | | | | | | | yes | no |
| 6 | Do health care providers provide AYFRH services in a non-judgmental, caring and supportive? | | | | | | | | | | | | | | | | | | | | | | yes | no |
| 7 | Does the health facility have Standard Operating Procedures (SOPs) explaining how the service outlets are kept clean, comfortable and attractive to adolescents & youth? | | | | | | | | | | | | | | | | | | | | | | yes | no |
| 8 | Do the consultation rooms for adolescent & youth ensure | | | | | | | | | | | | | | | | | | | | | | yes | no |
|  | 1. privacy (visual & auditory)? | | | | | | | | | | | | | | | | | | | | | | yes | no |
|  | 2. confidentiality (records locked and not accessible to other people)? | | | | | | | | | | | | | | | | | | | | | | yes | no |
| 9 | Are adequate amounts of the following drugs or supplies available in this facility? | | | | | | | | | | | | | | | | | | | | | | yes | no |
|  | 1. Male condoms | yes | | | | | no | | | | | 3 | | | | | | Oral contraceptives | | | | | yes | no |
|  | 2. Female condoms | yes | | | | | no | | | | | 4 | | | | | | emergency contraceptives | | | | | yes | no |
| 10 | Are the following services available in this health facility | | | | | | | | | | | | | | | | | | | | | | yes | no |
|  | 1. Emergency contraception | | | yes | | | | | no | | | | | 3 | | | | | | | HIV counseling and testing | | yes | no |
|  | 2. Safe abortion services | | | yes | | | | | no | | | | | 4 | | | | | | | Pregnancy test | | yes | no |
| 11 | Have you had shortages/stock outs of any of the drugs or supplies mentioned in Q 9 above in the last one month? If yes, which drugs or supplies | | | | | | | | | | | | | | | | | | | | | | yes | no |
|  | 1. Male condoms | | yes | | | | | no | | | | | 3 | | | | | | | Oral contraceptives | | | yes | no |
|  | 2. Female condoms | | yes | | | | | no | | | | | 4 | | | | | | | emergency contraceptives | | | yes | no |
| 12 | Does the health facility have IEC/BCC materials on the different components of AYFRH? | | | | | | | | | | | | | | | | | | | | | | yes | no |
| 13 | Does the health facility have guidelines/teaching materials for peer education? | | | | | | | | | | | | | | | | | | | | | | yes | no |
| 14 | Do staff of this health facility have adequate knowledge and skills to train adolescents & youth ? | | | | | | | | | | | | | | | | | | | | | | yes | no |
| 15 | Has this facility trained adolescent & youth peer educators in the last three months? how many?--- | | | | | | | | | | | | | | | | | | | | | | yes | no |
| 16 | Has support staff been given orientation on “Adolescent & Youth Friendly Services”? | | | | | | | | | | | | | | | | | | | | | | yes | no |
| 17 | Does the health facility have the case management guidelines | | | | | | | | | | | | | | | | | | | | | | yes | no |
|  | 1. STIs | | | | yes | | | | | no | | | | | | 4 | | | | | | Contraception/family planning | yes | no |
|  | 2. HIV/AIDS | | | | yes | | | | | no | | | | | | 5 | | | | | | Antenatal, delivery, postnatal | yes | no |
|  | 3. Safe abortion services | | | | yes | | | | | no | | | | | |  | | | | | |  | yes | no |
| 18 | Have staff been trained on the case management guidelines? If yes, which of the services? | | | | | | | | | | | | | | | | | | | | | | yes | no |
|  | 1. STIs | | | | | yes | | | | | no | | | | | | 4 | | Contraception/family planning | | | | yes | no |
|  | 2. HIV/AIDS | | | | | yes | | | | | no | | | | | | 5 | | Antenatal, delivery, postnatal | | | | yes | no |
|  | 3. Safe abortion services | | | | | | | | | | | | | | | | | | | | | | yes | no |
| 19 | Does the health facility have a functional referral and feedback (back referral) system | | | | | | | | | | | | | | | | | | | | | | yes | no |
|  | 1. Referral (one way) | | | | | | | | | | | | | | | | | | | | | | yes | no |
|  | 2. Feedback (back referral) | | | | | | | | | | | | | | | | | | | | | | yes | no |
| 20 | Are adolescents & youth trained in the provision of certain services? If yes types of services----- | | | | | | | | | | | | | | | | | | | | | | yes | no |
| 21 | Are adolescents & youth involved in planning/ implementation/, monitoring of AYFRH services? | | | | | | | | | | | | | | | | | | | | | | yes | no |

**2. HEALTH CARE PROVIDER INTERVIEW**

Sex of respondent:--------- Age------- Duration of service in this facility------

| Sn | Questions | | | | | | | | | | | | | Responses | | |
| --- | --- | --- | --- | --- | --- | --- | --- | --- | --- | --- | --- | --- | --- | --- | --- | --- |
|  |  |  |  |  |  |  |  |  |  |  |  |  |  | yes | no | dk |
| 1 | Have you used / referred / documents (Show the Documents) | | | | | | | | | | | | | yes | no |  |
|  | 1.Standards on Youth Friendly Reproductive Health Services | | | | | | | | | | | | | yes | no |  |
|  | 2.Tools for planning implementation and monitoring of YFS in the last one months | | | | | | | | | | | | | yes | no |  |
| 2 | Have you participated in delivering information on adolescent and youth reproductive rights and needs during the last one month in | | | | | | | | | | | | | yes | no |  |
|  | 1. Schools | yes | no | 3 | | | Community meetings ( Idir, Kebele meetings) | | | | | | | yes | no |  |
|  | 2. Youth centers | yes | no | 4 | | | Adolescent and/or youth meetings | | | | | | | yes | no |  |
| 3 | Have you been trained in YFS? | | | | | | | | | | | | | yes | no |  |
| 4 | Does the health facility have SOPs to keep service outlets clean, comfortable ,attractive | | | | | | | | | | | | | yes | no |  |
| 5 | Do the consultation rooms for adolescent & youth ensure: | | | | | | | | | | | | |  |  |  |
|  | 1. privacy (visual & auditory)? | | | | | | | | | | | | | yes | no |  |
|  | 2. confidentiality? (records locked and not accessible to other people) | | | | | | | | | | | | | yes | no |  |
| 6 | Are the medicines /supplies to manage adolescent & youth clients available in facility? | | | | | | | | | | | | | yes | no |  |
| 7 | Are the equipment to manage adolescents and youth clients available in this facility? | | | | | | | | | | | | | yes | no |  |
| 8 | In the last one month, have you had shortages of drugs and supplies If yes, explain__ | | | | | | | | | | | | | yes | no |  |
| 9 | In the last three months, has unavailability of equipment or nonfunctioning equipment? | | | | | | | | | | | | | yes | no |  |
| 10 | Are IEC/BCC materials currently available on the different components of AYRHS | | | | | | | | | | | | | yes | no |  |
|  | 1. Sexually transmitted infections/HIV/AIDS | | | | | | | | | | | | | yes | no |  |
|  | 2. Unwanted/unplanned pregnancy and contraceptive use/ family planning | | | | | | | | | | | | | yes | no |  |
|  | 3. Maternal health care (Antenatal Care, Delivery Care Postnatal Care) | | | | | | | | | | | | | yes | no |  |
|  | 4. Safe abortion services | | | | | | | | | | | | | yes | no |  |
| 11 | Does the health facility have case management guidelines for the following services? | | | | | | | | | | | | | yes | no |  |
|  | 1. STIs | | | | yes | | | | no | | 4 | | Contraception/family planning | yes | no |  |
|  | 2. HIV/AIDS | | | | yes | | | | no | | 5 | | Antenatal, delivery, postnatal | yes | no |  |
|  | 3. Safe abortion services | | | | yes | | | | no | |  | |  | yes | no |  |
| 12 | Have you been trained on the case management guidelines? If yes, which? | | | | | | | | | | | | | yes | no |  |
|  | 1. STIs | | | | | yes | | no | | 4 | | Contraception/family planning | | yes | no |  |
|  | 2. HIV/AIDS | | | | | yes | | no | | 5 | | Antenatal, delivery, postnatal | | yes | no |  |
|  | 3. Safe abortion services | | | | | yes | | no | |  | |  | | yes | no |  |
| 13 | Do you use the case management guidelines? | | | | | | | | | | | | | yes | no |  |
| 14 | Do you receive regular guidance on psychological, physical assessment and individualized care on adolescent & youth health services? If yes, how frequently?--- | | | | | | | | | | | | | yes | no |  |
| 15 | Does the health facility have referral formats/forms for adolescents& youth? | | | | | | | | | | | | | yes | no |  |
|  | 1. Referral (one way only) | | | | | | | | | | | | | yes | no |  |
|  | 2. Referral and Feedback (back referral) | | | | | | | | | | | | | yes | no |  |
| 16 | Do you use the referral forms when referring adolescents & youth to other service? | | | | | | | | | | | | | yes | no |  |
| 17 | Are adolescents & youth trained in the provision of certain services? | | | | | | | | | | | | | yes | no |  |
|  | If yes on what types of services have they been trained in? ------- | | | | | | | | | | | | |  |  |  |
| 18 | Are youth involved in providing health service to adolescents in the community? | | | | | | | | | | | | | yes | no |  |
|  | If so, could you describe what they do?---- | | | | | | | | | | | | |  |  |  |

**3. ADOLESCSENT & YOUTH CLIENT EXIT INTERVIEW**

| Sn | Questions | Response | |
| --- | --- | --- | --- |
|  |  |  | |
| 1 | Did you see a sign post containing information on: | yes | no |
|  | 1. the types of Adolescent and Youth Reproductive Health services provided | yes | no |
|  | 2. the working days and hours of the health facility | yes | no |
| 2 | Did you receive information on the available SRH services in this health facility? | yes | no |
| 3 | Did the health-care provider listen to what you said with interest? | yes | no |
| 4 | Did the health-care provider treat you in a supportive and considerate manner? | yes | no |
| 5 | Did you find the health facility | yes | no |
|  | 1.Clean? | yes | no |
|  | 2. Comfortable? | yes | no |
|  | 3. Attractive? | yes | no |
| 6 | Do you believe that |  |  |
|  | 1. others could hear your discussion with the health-care provider | yes | no |
|  | 2.others see your consultation with the care provider? | yes | no |
| 7 | Do you believe the information you provided is kept in secret (confidential)? | yes | no |
| 8 | Did you get medicines and supplies for you at this facility? | yes | no |
| 9 | Was their educational materials about ASRH displayed at this facility? | yes | no |
| 10 | Did the materials contain information that you found useful? | yes | no |
| 11 | Was the health care provider considerate and respectful? | yes | no |
| 12 | Was s/he critical of any of your words or actions? | yes | no |
| 13 | Did the provider take time to listen, do necessary examination, deliver the services? | yes | no |
| 14 | Did the health-care provider refer you to another place? | yes | no |
|  | 1.If so, did he/she explain to you why you were referred? | yes | no |
|  | 2. If so, did he/she explain where you were referred to? | yes | no |
| 15 | Will you recommend the health facility for others | yes | no |

**4. OBSERVATION AND RECORD REVIEW CHECKLIST**

| Sn | Observation or Record Review | | | | | | | | Available | | | Explanations/Remarks |
| --- | --- | --- | --- | --- | --- | --- | --- | --- | --- | --- | --- | --- |
|  |  |  |  |  |  |  |  |  | Yes | No | |  |
| 1 | AYRH relevant documents | | | | | | | | yes | no | | Specify other relevant documents |
|  | 1. AYRH Strategy | | | | | | | | yes | no | |  |
|  | 2. YFS Standards and Minimum Service Delivery Package | | | | | | | | yes | no | |  |
|  | 3. Tools for planning, Implementation & monitoring AYFRH | | | | | | | | yes | no | |  |
|  | 4.SOPs related to AYFRHS | | | | | | | | yes | no | |  |
| 2 | Sign post information shown on signpost | | | | | | | | yes | no | |  |
|  | 1. the types of SRH services provided | | | | | | | | yes | no | |  |
|  | 2. the working days and hours | | | | | | | | yes | no | |  |
|  | 3. it is in good condition and is displayed in a prominent location | | | | | | | | yes | no | |  |
| 3 | IEC materials at the waiting areas | | | | | | | | yes | no | |  |
|  | 1. Sexually transmitted infections/HIV/AIDS | | | | | | | | yes | no | |  |
|  | 2.Unwanted/unplanned pregnancy and contraceptive use/FP | | | | | | | | yes | no | |  |
|  | 3. Maternal health care (ANC, Delivery Care Postnatal Care) | | | | | | | | yes | no | |  |
|  | 4. Safe abortion services | | | | | | | | yes | no | |  |
| 4 | Plan for the provision of AYFRH information and services at schools, youth centers and community organizations ( Eg Kebele meetings, Idir) | | | | | | | | yes | | no | Check records to see the plan |
| 5 | Provision of AYFRH information and services at schools, youth centers and community organizations ( Eg Kebele meetings, Idir meetings ) | | | | | | | | yes | | no | Check records to see services were provided |
| 6 | Cleanliness and attractiveness of the facility   1. ***General cleanliness at the following:*** | | | | | | | |  | |  | Cleanliness means that there is no dirt, no litter, and no bad smell |
|  | 1. Surrounding | | yes | | no | | 4 | Waiting room | yes | | no |  |
|  | 2.Reception Counter | | yes | | no | | 5 | Consultation room | yes | | no |  |
|  | 3. Examination room | | yes | | no | | 6 | Toilets | yes | | no |  |
|  | 1. Availability of ***the following:*** | | | | | | | | yes | | no | Observe drainage system, segregation of wastes, adequate number of waste bins, cleaning tools, detergents, and availability of running water |
|  | 1. Good drainage | | | | | | | | yes | | no |  |
|  | 2. Covered waste bins | | | | | | | | yes | | no |  |
|  | 3. Running water | | | | | | | | yes | | no |  |
|  | 4. Cleaning tools (broom, scrub, brush, cloths etc.) | | | | | | | | yes | | no |  |
|  | 5. Disinfectants/detergents | | | | | | | | yes | | no |  |
| 7 | Provision of privacy and confidentiality | | | | | | | | yes | | no | Check availability of separate room |
|  | 1.Separate room to ensure both auditory and visual privacy | | | | | | | | yes | | no |  |
|  | 2.possibility of hearing conversations b/n health provider and client | | | | | | | | yes | | no | possibility of hearing |
| 8 | Availability of drugs, supplies and services | | | | | | | | yes | | no |  |
|  | 1. Condoms | yes | | no | | 4 | Safe abortion services | | yes | | no |  |
|  | 2. Oral contraceptives | yes | | no | | 5 | HIV test | | yes | | no |  |
|  | 3.Emergency contraceptives | yes | | no | | 6 | Pregnancy test | | yes | | no |  |
| 9 | Availability of referral format/forms | | | | | | | | yes | | no |  |
| 10 | Use of referral forms | | | | | | | | yes | | no | Review filled forms |
| 11 | Plan for training of adolescents and youth as peer educators | | | | | | | | yes | | no | review documents |
| 12 | Involvement of adolescents and youth | | | | | | | | yes | | no | Ask /review documents |

***5. CLIENT-PROVIDER INTERACTION OBSERVATION***

| Sn | Quality assessment question | | | | | | | | | Response | |
| --- | --- | --- | --- | --- | --- | --- | --- | --- | --- | --- | --- |
| 1 | What was the reason for the consultation? | | | | | | | | |  | |
| 2 | Was anyone else present in the room at the time of consultation? | | | | | | | | | 1.yes | 2.no |
| **3** | **At the beginning of the consultation did the health-care provider:** | | | | | | | | |  |  |
| 1 | Seat adolescent in the position that facilitated communication most easily? | | | | | | | | | 1.yes | 2.no |
| 2 | Introduce himself/herself first to the adolescent? | | | | | | | | | 1.yes | 2.no |
| 3 | Ask the adolescent who he/ she has brought with him/her to the consultation? | | | | | | | | | 1.yes | 2.no |
| 4 | Show interest in the adolescent and spend some time getting to know him/ her before focusing on the medical problems (problem free talk)? | | | | | | | | | 1.yes | 2.no |
| 5 | Did anyone else enter the room during the consultation? | | | | | | | | | 1.yes | 2.no |
| 6 | Did the service provider listen with attention to what the client had to say? | | | | | | | | | 1.yes | 2.no |
| 7 | Did the service provider assure the client that no information will be disclosed to anyone (parents/other) without their permission? | | | | | | | | | 1.yes | 2.no |
| 4 | Did the service provider deny any services to this adolescent/young client? | | | | | | | | | 1.yes | 2.no |
| 1 | Unavailable in the facility | | 1.yes | | 2.no | | 4 | | Inability to pay | 1.yes | 2.no |
| 2 | Age below 18 | 1.yes | | 2.no | | 5 | | The condition needs referral | | 1.yes | 2.no |
| 3 | Unmarried | 1.yes | | 2.no | | 6 | | Other (please specify) | | 1.yes | 2.no |
| 5 | During the consultation did the service provider take any psychosocial history, such as: | | | | | | | | | | |
| 1 | Asked the adolescent questions about home and relationships with adults? | | | | | | | | | 1.yes | 2.no |
| 2 | Asked the adolescent questions about school? | | | | | | | | | 1.yes | 2.no |
| 3 | Asked the adolescent questions about sexual relationships? | | | | | | | | | 1.yes | 2.no |
| 4 | Asked the adolescent questions about smoking, alcohol or other substances? | | | | | | | | | 1.yes | 2.no |
| 5 | If an informed consent from a third party was required, was adolescent assent to the service/procedure also obtained? | | | | | | | | | 1.yes | 2.no |
| 6 | During the consultation did the service provider do the following: | | | | | | | | |  |  |
| 1 | Talk about how to prevent diseases, and what to do to stay healthy? | | | | | | | | | 1.yes | 2.no |
| 2 | Inform the adolescent client about the services available for him/her? | | | | | | | | | 1.yes | 2.no |
| 3 | Provide accurate and clear information on the medical condition? | | | | | | | | | 1.yes | 2.no |
| 4 | Provide accurate and clear information on the management/treatment options? | | | | | | | | | 1.yes | 2.no |
| 6 | Provide accurate and clear information on follow-up actions? | | | | | | | | | 1.yes | 2.no |
| 8 | Ask the adolescent client whether he/she has any problem understanding the treatment that is being provided? | | | | | | | | | 1.yes | 2.no |
| 9 | Check the adolescent client’s understanding of the information provided by asking probing questions? | | | | | | | | | 1.yes | 2.no |
| 10 | Use audio-visual material to explain anatomy, disease, or other, as relevant to the topic of the consultation? | | | | | | | | | 1.yes | 2.no |
| 11 | Ask the adolescent client’s permission before performing the examination/procedure? | | | | | | | | | 1.yes | 2.no |
| 12 | Explain the results of the physical examination to the client | | | | | | | | | 1.yes | 2.no |
| 7 | Did the service provider refer the adolescent client to another health facility? | | | | | | | | | 1.yes | 2.no |

**Guide for Qualitative data collection**

1. Age-------
2. Sex--------
3. year of service-------
4. How do you explain the SRH service utilization in the facility(in the zone for officers)- prove
5. How do you explain the current approach (age driven approach)? Prove (advantage? disadvantage)in terms of addressing the sexual and reproductive health need of young population?
6. Is there a challenge to address the SRH need of young population? Explain
7. What should be done to improve the service?
